# Low Vision Rehabilitation in Older Adults

**DOI:** 10.4274/tjo.68878

**Published:** 2016-06-06

**Authors:** Zuhal Özen Tunay, Aysun İdil, İkbal Seza Petriçli, Özdemir Özdemir

**Affiliations:** 1 Zekai Tahir Burak Women’s Health Training and Research Hospital, Ophthalmology Clinic, Ankara, Turkey; 2 Ankara University Faculty of Medicine, Department of Ophthalmalogy, Low Vision Rehabilitation and Research Center, Ankara, Turkey; 3 Zübeyde Hanım Women’s Health Training and Research Hospital, Ophthalmology Clinic, Ankara, Turkey

**Keywords:** Geriatric, low vision, low vision rehabilitation

## Abstract

**Objectives:**

To evaluate the diagnosis distribution, low vision rehabilitation methods and utilization of low vision rehabilitation in partially sighted persons over 65 years old.

**Materials and Methods:**

One hundred thirty-nine partially sighted geriatric patients aged 65 years or older were enrolled to the study between May 2012 and September 2013. Patients’ age, gender and the distribution of diagnosis were recorded. The visual acuity of the patients both for near and distance were examined with and without low vision devices and the methods of low vision rehabilitation were evaluated.

**Results:**

The mean age of the patients was 79.7 years and the median age was 80 years. Ninety-six (69.1%) of the patients were male and 43 (30.9%) were female. According to the distribution of diagnosis, the most frequent diagnosis was senile macular degeneration for both presenile and senile age groups. The mean best corrected visual acuity for distance was 0.92±0.37 logMAR and 4.75±3.47 M for near. The most frequently used low vision rehabilitation methods were telescopic glasses (59.0%) for distance and hyperocular glasses (66.9%) for near vision. A significant improvement in visual acuity both for distance and near vision were determined with low vision aids.

**Conclusion:**

The causes of low vision in presenile and senile patients in our study were similar to those of patients from developed countries. A significant improvement in visual acuity can be achieved both for distance and near vision with low vision rehabilitation in partially sighted geriatric patients. It is important to guide them to low vision rehabilitation.

## INTRODUCTION

According to the World Health Organization’s VISION 2020 report, the prevention and rehabilitation of low vision is among the primary global objectives. The legal definitions of ‘low vision’ and ‘blindness’ specified by the World Health Organization are based on visual acuity and visual field. Low vision is defined as visual acuity in the better eye after refractive correction between 20/70 (0.3) and 20/400 (0.05, 3 mps) or a visual field less than 20 degrees.^1,2^ In low vision, the degree of vision loss is less than in blindness and the individual benefits from vision enhancement aids.^3,4^

Low vision significantly impacts a person’s quality of life and is a major socioeconomic problem for both individuals and the public. As the elderly population increases and age-related vision problems become more common, the importance of low vision rehabilitation is also growing. Due to demographic, socioeconomic and cultural differences, it is important that each community investigate the distribution of diagnoses, determine preventable causes and use these data in health planning for its own population.^3,5,6^

The aim of this study was to evaluate and contribute to our national body of knowledge regarding the causes of low vision, methods of vision enhancement, and utilization of low vision rehabilitation in presenile and senile individuals presenting for low vision rehabilitation.

## MATERIALS AND METHODS

One hundred thirty-nine patients aged 65 years or older who presented to our clinic for the first time between May 2012 and September 2013 were enrolled in the study. A detailed medical history was obtained from each partially sighted subject and the areas in which they experienced difficulty due to near and distance visual function were identified. Near and distance best corrected visual acuity (BCVA) were determined. Color vision and intraocular pressure were evaluated and anterior and posterior examinations were conducted. Subjects were evaluated in terms of age at presentation, gender, distribution of diagnoses in the presenile (65-74 years old) and senile (75 years and older) age groups, near and distance visual acuity, and low vision aids (LVAs) used for near and distance.

All subjects’ near and distance visual acuity were determined after correcting refractive errors. Distance vision was measured using the ETDRS chart from 4, 2 or 1 meter(s) depending on the subject’s vision level and was recorded in logMAR. Near vision was determined using the MNREAD near reading chart and vision levels from 25 cm were reported as “M” values.

The patients were asked about their priorities for low vision rehabilitation. Low vision enhancement devices used were Keplerian and Galilean telescopic and electro-optical systems for distance and magnifiers, hyperocular lenses, labo-clip glasses, telemicroscopes and electro-optical systems for near vision.

Considering the patients’ visual acuity, visual field analysis, binocular vision status, and visual needs and expectations, required magnification power was calculated with the Kestenbaum formula and appropriate low vision enhancement methods were determined. Patients’ visual acuity using the LVA was assessed and they were informed on the use of the LVA.

The study was conducted in accordance with the Declaration of Helsinki and was approved by the Ankara University Ethics Committee. Informed consent was obtained from all study participants.

SPSS for Windows version 16.0 (Statistical Package for the Social Sciences Inc., Chicago, IL, USA) software was used for statistical analyses. Data are presented as minimum (min), maximum (max), mean, standard deviation (SD), number (n) or percentage (%). Vision levels with and without LVA use were compared using a paired-samples t test. The level of significance was accepted as ɑ=0.05.

## RESULTS

The mean age of the patients was 79.7 years (65-101 years), the median age was 80; 69.1% (n=96) were male and 30.9% (n=43) were female.

The mean BCVA of the better eye was 0.92±0.37 (0.20-1.60) logMAR for distance and 4.75±3.47 (1.00-16.00) M for near vision.

The most common diagnosis was age-related macular degeneration (Table 1).

The priority of the patients presenting for low vision rehabilitation was to improve their near vision; 62.5% (87 patients) stated that they required low vision rehabilitation primarily for near vision, while 37.5% expressed that they had more difficulty with distance vision.

The distribution of LVAs chosen for near and distance vision is shown in Tables 2 and 3. Spectacles alone provided adequate improvement in distance vision for 47 patients (33.8%), while 92 (66.2%) of the patients were prescribed an LVA. Telescopic lenses were the most common method (59.0%) chosen for distance. Of the 92 patients who did not achieve adequate distance vision with conventional glasses and were prescribed an LVA, 89% (82 patients) used telescopic lenses.

A total of 182 LVAs for near vision were prescribed for the 139 patients in the study; 30.9% of the study participants used more than one LVA. Hyperocular glasses were the most common LVA used for near vision (66.9%). Of the patients using spectacles as an LVA for near, 30.9% were also prescribed a magnifier for specific daily activities.

The near and distance vision levels attained by the study participants using LVAs are shown in Table 4. Mean distance vision improved from 0.92 logMAR to 0.24 logMAR and near vision improved from 4.75 M to 1.44 M with LVA use. The differences were significant for both near and distance (paired-samples t test, p=0.001).

At 1 year follow-up, 91.4% (n=127) of the patients reported that they continued to use the LVA. Of the 12 patients (8.6%) who did not continue LVA use, it was determined that further decline in vision level due to underlying ophthalmic pathology necessitated a new LVA system.

## DISCUSSION

Aging is a physiological process that affects every system of the body. With longer life expectancy and the resulting rise in the elderly population, old age has increasing importance as a physiological stage of life. Vision is one of the functions most severely affected in the geriatric age group.^7^ This study investigated the clinical characteristics and low vision rehabilitation methods applied in low vision patients in the senile and presenile groups.

The advantage of this type of study conducted on individuals presenting to clinics is the more reliable and detailed ophthalmologic data included.^8^ However, the main disadvantage is that the data cannot be generalized to the general public. The Ankara University Low Vision Rehabilitation and Research Center is a university-based center that serves patients from every region of Turkey. Therefore, these data should contribute both in terms of referring patients with low vision to rehabilitation services and to the planning and implementation of low vision rehabilitation services.

There was quite a wide age range among the patients presenting for low vision rehabilitation, with the oldest being 101 years old. We believe this demonstrates that there is no age limit for low vision rehabilitation in the senile group and that individuals at every age have the potential for low vision rehabilitation depending on their individual needs and expectations. Data from Western countries show similar wide age ranges, whereas data from developing countries indicates that low vision rehabilitation services are not widespread and a higher proportion of patients are in the presenile age group.^5,6,9,10^

Consistent with gender distributions reported in the literature, there were more males (69.1%) presenting in this age group.^9, 10, 11^ This may be attributable to two factors. First, in society men may have greater need for visual function for economical and social reasons; second, men may have fewer esthetic concerns and may therefore be more willing to use low vision enhancement methods.

The most common diagnosis in our study group was age-related macular degeneration. The second and third most common diagnoses were diabetic retinopathy and hereditary retinal disease in the presenile group versus glaucoma-related vision loss and diabetic retinopathy in the senile group. In a 2008 study, Recep et al.^12^ reported that among all age groups, 22% of patients were enrolled in the low vision rehabilitation program with a diagnosis of age-related macular degeneration, and a high proportion of patients with this diagnosis benefited from telescopic lenses. In Western countries, the diagnostic groups most commonly requiring low vision rehabilitation in the senile group are age-related macular degeneration and diabetic retinopathy. In contrast, in developing countries, the diagnosis distribution of this age group is dominated by patients requiring cataract surgery.^8,9,10^

For distance vision, spectacles alone provided sufficient improvement for 33.8% of our patients. This indicates that accurately determining refractive error and current visual function is one of the most crucial steps in a low vision examination.^11,13,14 ^ Furthermore, the small increase in visual acuity provided by the complete and accurate correction of refractive errors may result in lighter and more effective LVAs for the patient and increase their use of the device.^4^

Among all the patients in our study, the LVA most frequently used for distance was telescopic glasses (59%). Among patients prescribed an LVA for distance vision, this rate was 89%. These systems are preferred because they are portable and more economical than electro-optical systems. The main disadvantage of telescopic glasses is that they may be difficult for some individuals to accept due to esthetic concerns. However, the utilization rate increases in the presenile and senile age groups as people in this group are generally less concerned with appearances. In the literature, the most commonly utilized LVA is telescopic glasses. In Turkey, Petriçli et al.^11^ reported that 70% of patients in the low vision rehabilitation group used telescopic glasses. In a study by Altınbay^15^ 74% of the patients were prescribed telescopic glasses, although only 54% reported purchasing them. Recep et al.^12^ and Bakbak et al.^16^ reported that all of the patients in their studies used telescopic glasses. Compared to data from Western countries, our study indicates that electro-optical systems were less commonly used. The higher cost of electro-optical systems compared to telescopic systems is the biggest reason they are not utilized more in low vision rehabilitation.

We determined that improving near vision was the priority of most patients presenting for low vision rehabilitation. The most common LVA for near vision in our study was hyperocular eyeglasses (followed by telemicroscopes and labo-clip glasses), consistent with the literature.^9,11^ Using magnifiers is not the first choice among this age group, and they are prescribed to many patients as an auxiliary LVA especially for certain daily activities and reading.^9,11,14^ In the current study, 30.6% of the patients needed more than one type of LVA for near vision. We believe that it is important to keep in mind that individuals may require more than one low vision rehabilitation method for their daily activities such as reading, cooking, and self-care needs.

After 1 year, 91.4% of the patients in our study reported that they were still using their LVA. In the few patients who did not continue using the LVA, we determined that further decline in vision level due to underlying ophthalmic pathology necessitated a new LVA system. The reason for our high compliance rate compared to previous studies may be that our patients began using the LVAs after being trained in their use, and frequent follow-up maintained high motivation.^17^ This reinforces the fact that low vision rehabilitation is not limited to LVA utilization, but should entail adaptations that encompass all aspects of the patient’s life.

In summary, low vision rehabilitation may be necessary in geriatric patients due to serious ophthalmologic and neurologic problems. Optimizing the visual abilities of partially sighted patients makes their day-to-day lives easier, increases life quality, and allows them to continue to be self-sufficient, productive and independent individuals. Therefore, we would like to emphasize the importance of referring partially sighted patients to low vision rehabilitation during the course of their clinical follow-up.

## Ethics

Ethics Committee Approval: Ankara University Ethics Committee, Informed Consent: It was taken.

Peer-review: Externally peer-reviewed.

## Figures and Tables

**Table 1:**
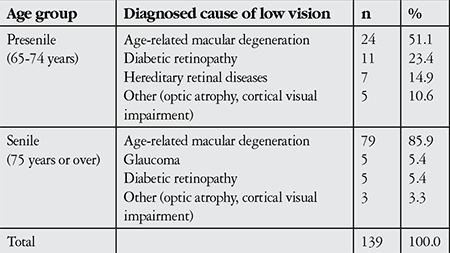
Diagnosis distribution in the presenile and senile age groups

**Table 2:**
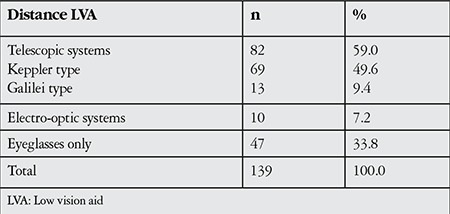
Low vision aids used for distance vision

**Table 3:**
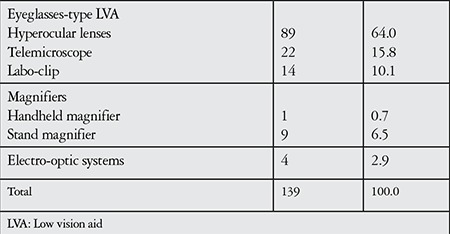
Low vision aids used for near vision

**Table 4:**
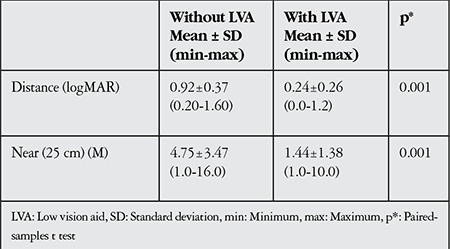
Comparison of patients’ visual acuity with and without low vision aids
